# Repressive and non-repressive chromatin at native telomeres in *Saccharomyces cerevisiae*

**DOI:** 10.1186/1756-8935-2-18

**Published:** 2009-12-02

**Authors:** Esther R Loney, Peter W Inglis, Sarah Sharp, Fiona E Pryde, Nicholas A Kent, Jane Mellor, Edward J Louis

**Affiliations:** 1 Department of Oncology, University of Western Ontario, Ontario, Canada; 2 Embrapa Recursos Geneticos e Biotecnologia, Parque Estacao Biologica, Brasilia-DF, CEP 70770900, CP, 02372, Brazil; 3 Institute of Genetics, University of Nottingham, Nottingham, UK; 4 University of Edinburgh, WTCCB, Edinburgh, UK; 5 Cardiff School of Biosciences, Cardiff University, Cardiff, UK; 6 Department of Biochemistry, University of Oxford, Oxford, UK

## Abstract

**Background:**

In *Saccharomyces cerevisiae *genes that are located close to a telomere can become transcriptionally repressed by an epigenetic process known as telomere position effect. There is large variation in the level of the telomere position effect among telomeres, with many native ends exhibiting little repression.

**Results:**

Chromatin analysis, using microccocal nuclease and indirect end labelling, reveals distinct patterns for ends with different silencing states. Differences were observed in the promoter accessibility of a subtelomeric reporter gene and a characteristic array of phased nucleosomes was observed on the centromere proximal side of core X at a repressive end. The silent information regulator proteins 2 - 4, the yKu heterodimer and the subtelomeric core X element are all required for the maintenance of the chromatin structure of repressive ends. However, gene deletions of particular histone modification proteins can eliminate the silencing without the disruption of this chromatin structure.

**Conclusion:**

Our data identifies chromatin features that correlate with the silencing state and indicate that an array of phased nucleosomes is not sufficient for full repression.

## Background

Heterochromatin is defined as regions of DNA that remain highly condensed throughout the cell cycle. Although, yeast chromosomes are too small to visualize condensed chromatin, several regions of the *S. cerevisiae *genome show similarities to the heterochromatin of higher organisms [1,2]. The silent mating-type loci, *HML *and *HMR*, the tandem rDNA array and regions close to telomeres, in particular, exhibit heterochromatic properties, such as position effects on gene expression and chromatin that is less accessible to restriction enzymes and DNA methylases [3-5].

Transcriptional silencing at telomeres and the silent mating-type loci is dependent on the silent information regulator proteins 2 - 4 (Sir2 - 4), which are integral components of the silenced chromatin. The Sir proteins interact with each other and with hypoacetylated histones H3 and H4 to form a repressive structure. At *HML *and *HMR *the Sir complex is recruited by Rap1, Abf1 and the origin recognition complex (ORC) which bind to the *HM *silencers. At telomeres the Sir complex is recruited by Rap1 bound to the telomeric repeats. Once recruited, Sir2 is thought to deacetylate the histones of adjacent nucleosomes allowing the Sir complex to spread outwards from the site of assembly [6,7]. The presence of other histone modifications, such as histone variant H2A.Z, methylation of lysine residues 4 and 79 of histone H3 and the acetylation of lysine 16 in histone H4, may limit the spread of the Sir complex [8-12].

The multiple interactions formed among the Sir proteins and histones are thought to create an inaccessible chromatin structure resulting in silencing [3-5]. At both *HM *loci nucleosomes are arranged in regularly spaced arrays, in contrast to the less ordered structure at the expressed *MAT *locus [13,14], and this structure is dependent on the Sir proteins [13,14]. Similarly, at a truncated telomere, which lacks all of the subtelomeric repeat elements, an array of phased nucleosomes is present in the region adjacent to the telosome [15]. This structure is consistent with silencing levels at truncated ends which diminish with distance from the telomeric repeats [16].

The regular chromatin structure observed at truncated telomeres, cannot explain certain features of silencing at native ends. First, the silencing at the native ends is discontinuous, with the greatest degree of silencing observed immediately adjacent to the telomeric repeats and around the subtelomeric core X element [17]. This is due to the presence of anti-silencing regions within the X associated repeats, which impede the spread of silencing at the native ends along with relay elements that can re-establish silencing discontinuously [18]. Secondly, while all truncation ends studied have exhibited strong silencing, the level of silencing varies among the native ends with many ends showing only weak repression [17]. If the chromatin structure is indicative of the silencing state we would expect to see differences in chromatin structure between native ends.

This study examines the chromatin structures of truncation, native repressive and native non-repressive telomeres in order to establish a link between the underlying chromatin structure and the silencing state of the telomeres. We also examine the roles of core X, the yKu and Sir proteins and certain chromatin modifying proteins (Bre1, Dot1, Set1, Sas2 and Bdf1) in the formation of the chromatin structure at native telomeres.

## Results

### The chromatin structure of native ends differs from truncated telomeres

**Figure 1:**
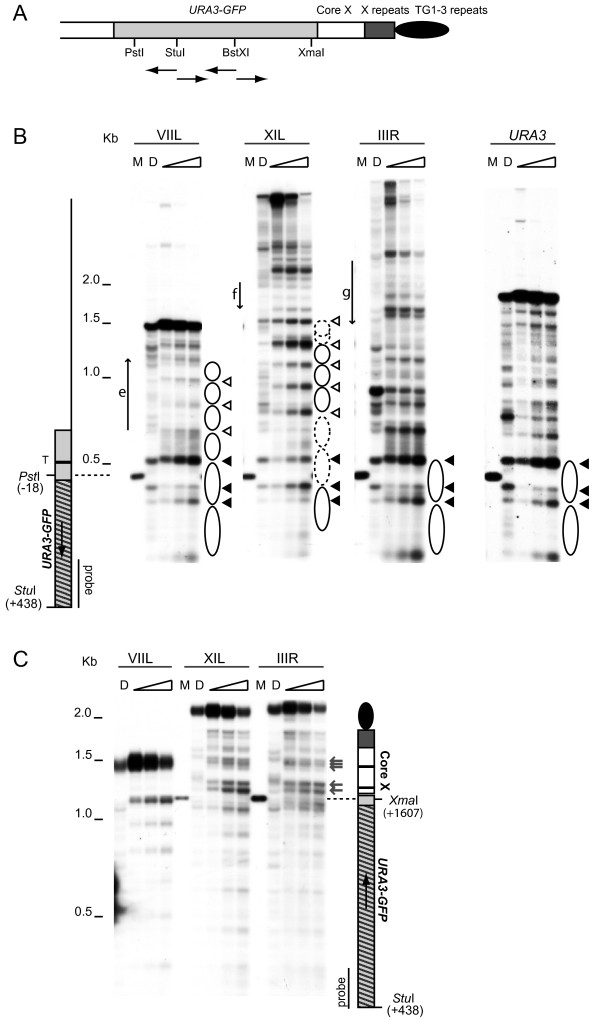
**Comparison of chromatin structures by indirect-end-label analysis at three telomeres of *Saccharoyces cerevisiae***. (A) Schematic of a *URA3-yEGFP *marked native telomere showing relevant restriction sites. Probes used for indirect end-labelling are indicated by arrows. (B) The subtelomeric chromatin structure of the truncated VIIL and native IIIR and XIL telomeres of *S. cerevisiae *was analysed by MNase digestion and indirect end labelling with the indicated probe. The chromatin structure of an isogenic strain containing the *URA3-yEGFP *construct at the native *URA3 *locus was also analysed. A control MNase digest of deproteinized DNA (D), and marker bands generated by digestion with *Stu*I and *Pst*I (M) are also shown. The position of the inserted *URA3-yEGFP *cassette is shown by a light grey box with hatching to indicate the *URA3-yEGFP *CDS; the TATA box (T) is indicated with a black bar. Restriction sites are numbered from the *URA3 *start codon. Three promoter-associated hypersensitive sites are indicated by black arrow heads and an array of evenly spaced hypersensitive sites, present at the VIIL and XIL telomeres, by white arrow heads. Inferred nucleosome positions are shown by ovals. The most telomere-proximal open reading frames, YGL256W (e), YKL224C (f), and YCR107W (g) are indicated by arrows to the left of each blot. (C) The chromatin structure downstream of the *URA3-yEGFP *reporter was detected using a probe on the telomere proximal side of the *Stu*I site. Marker bands were obtained by digestion with *Stu*I and *Xma*I. MNase hypersensitive sites adjacent to the core X binding sites are indicated by grey arrows. A schematic of the IIIR and XIL telomeres is shown with the core X ACS and Abf1 binding sites indicated by black bars. The truncation end VIIL is identical except that it lacks the core X and STR repeats.

Telomeres XIL and IIIR have previously been shown to be repressive and non-repressive ends, respectively [17]. In order to determine whether or not there is a correlation between subtelomeric chromatin structure and silencing state, the chromatin structure was analysed at these two native ends and a truncation end by digestion with microccocal nuclease (MNase). The strains used contain a *URA3-GFP *reporter construct, either adjacent to the core X element at telomeres IIIR or XIL or adjacent to the telomeric repeats at the truncated telomere VIIL (Figure 1A). Analysis of the subtelomeric chromatin structure upstream of the *URA3 *reporter (towards the centromere) of the truncated and native ends reveals several striking features (Figure 1B). The subtelomeric regions of both the truncated (VII-L) and native repressive (XIL) ends have a strong pattern of evenly spaced MNase hypersensitive sites (the white arrow heads in Figure 1B) consistent with a heterochromatic chromatin structure. In contrast, the subtelomeric region of the non-repressive IIIR telomere has an irregular MNase digestion pattern which is different from the deproteinized DNA control. The three bands closest to the *URA3 *promoter region (the black arrow heads in Figure 1B) have a different MNase sensitivity pattern at each telomere. At IIIR the pattern closely resembles the non-repressed promoter structure observed at the native *URA3 *locus with increased MNase accessibility compared to the deproteinized DNA digest (Figure 1B and [19]). However, at the repressive XIL telomere the sensitivity of all three sites is different from IIIR. In particular, the site closest to the TATA box (the top black arrow head in Figure 1B) shows a high level of protection, as it shows decreased cleavage both in relation to IIIR and to the deproteinized DNA sample. These results are consistent with the level of silencing observed at each end. The pattern of the promoter-proximal bands in the truncated telomere bears more resemblance to the non-repressive structure, despite the heterochromatic pattern towards the centromere. Truncated telomeres are known to switch between expressing and non-expressing states [20] which suggests that this MNase digestion pattern may arise from a mixed population of repressive and non-repressive chromatin structures.

In order to confirm that the chromatin structures of IIIR and XIL are characteristic of native ends, we analysed the chromatin structure of other telomeres (Additional File 1 and data not shown). The pattern of evenly spaced (phased) nucleosomes and a closed promoter was present at other repressive ends, demonstrating that the chromatin structure of XI left is characteristic of repressive ends in general. In contrast, each non-repressive end displayed an open promoter structure, similar to at IIIR, and had a unique MNase sensitivity pattern upstream of *URA3 *(Additional File 1 and Figure 1A). Further upstream (>500 bp) of the *URA3 *marker, all of the ends studied had stretches of regularly spaced MNase sensitive sites, which could be arrays of positioned nucleosomes, flanked by regions of enhanced sensitivity (Figure 1A and Additional File 1). A comparison of these regions with the *Saccharomyces *genome database suggests they are the locations of subtelomeric genes.

### The chromatin structure is not affected by the marker insertion

**Figure 2:**
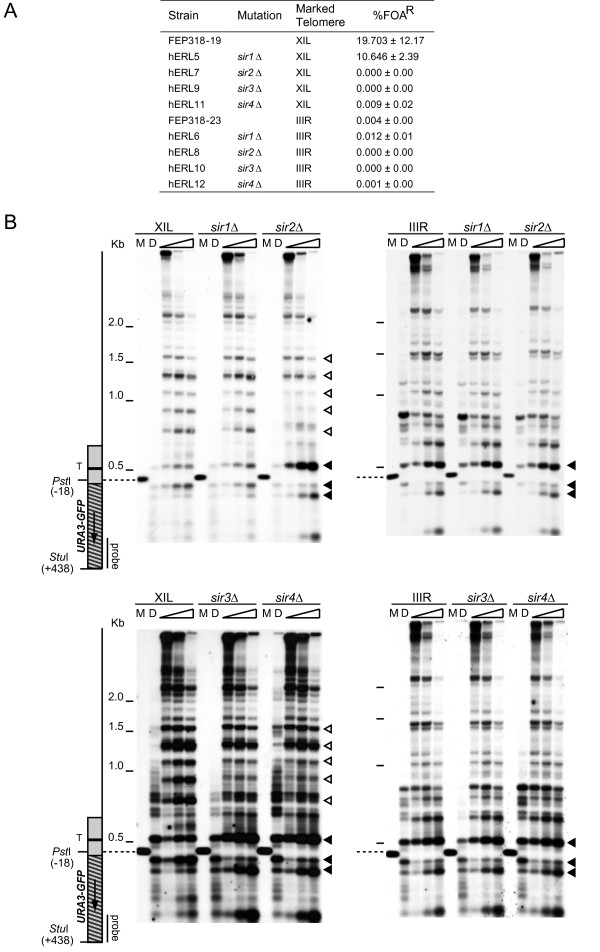
**The Sir complex is required for the formation of repressive chromatin at a native telomere**. (A) Frequency of fluoroorotic acid (FOA) resistance in isogenic Δ*sir::KanMX *strains containing the *URA3-yEGFP *marker adjacent to the core X element at the indicated telomere. The mean and standard deviation of FOA resistance is given for each strain. (B) Chromatin structures of the XIL and IIIR telomeres were analysed in Δ*sir1::KanMX*, Δ*sir2::KanMX*, Δ*sir3::KanMX *and Δ*sir4::KanMX *strains, by MNase digestion and indirect end labelling, as described for Figure 1B.

The phased array of MNase sensitive sites on the centromere proximal side of the *URA3 *marker at telomere XI left could be affected by the marker insertion itself. In order to assess this possibility, the region was assayed using markers integrated at varying distances from core X. As can be seen in Figure 2 and Additional File 2, the array of evenly spaced nucleosomes is intact and in the same location for insertions placed adjacent and up to 2 kb away from core X, showing limited alteration from the marker insertion. This supports the underlying assumption that integration of the *URA3 *construct does not alter the native chromatin structure. The lack of unique sequences and useful restriction sites within the region precludes an assessment without the marker.

### The structure of core X

In contrast to the chromatin differences observed between the ends upstream of the *URA3 *reporter, the chromatin patterns toward the telomere of the native ends are virtually identical (Figure 1C). Core X elements are found at all yeast telomeres; they contain an ARS consensus sequence (ACS) and, in most cases, a binding site for Abf1. At both XIL and IIIR, there were hypersensitive regions within core X near the ACS and Abf1 binding site (grey arrows Figure 1C). This pattern has been previously reported for the native IIIL telomere [21,22] and, has been interpreted as indicating regions of nucleosome exclusion due to the binding of ORC and Abf1. Our results indicate that the pattern over core X is unaffected by the degree of silencing at a particular end.

The rest of the region towards the telomere appears protected, with no strong nucleosomal banding pattern (Figure 1C), and the Y' element at XVR appears similarly protected (Additional File 2 and Figure 2C). The absence of a repressive pattern over the elements between core X and the telomere agrees with previous observations that this region is not silenced [17]. The pattern of digestion towards the telomere of the truncation end, which lacks core X, is dramatically different with a heterochromatin-like banding pattern persisting from within *URA3-yEGFP *through to the telosome (Figure 1C), as described previously [15]. The persistence of a strongly repressive chromatin structure towards the telomere is consistent with the continuous spread of silencing at truncated telomeres [16].

### Deletion of *SIR2*, *SIR3 *or *SIR4 *disrupts repressive chromatin

The arrangement of nucleosomes at the silent *HM *loci is dependent on the Sir proteins [13,14]. In order to determine whether the Sir proteins also influence the chromatin at both repressive and non-repressive telomeres, we deleted each *SIR *gene individually in our *URA3-GFP *marked strains. Deletion of *SIR2*, *SIR3 *or *SIR4 *abrogated silencing at XIL (Figure 2A) and dramatically altered the repressive chromatin structure of that telomere (Figure 2B left panels). In the *sir2-4 *mutants the MNase sensitivity pattern of the three promoter-associated bands (the black arrow heads in the left panels of Figure 2B) was very similar to the open promoter configuration observed at IIIR (the black arrow heads in the right panels in Figure 2B). Both the upper and lower bands displayed increased in intensity in the *sir2-4 *mutants than in the control strain and there was a decreased intensity in the middle promoter-associated band. The pattern of evenly spaced hypersensitive sites was also disrupted in the *sir2-4 *mutants (the white arrow heads in the left panels of Figure 2B). The intensity of these bands was decreased and there was a slight change in their spacing, suggesting that the nucleosomes that were present in this region had either been removed or become unphased. Further towards the centromere, the pattern of MNase digestion became indistinguishable from that of the control, Sir^+ ^strain, indicating that the affects of deleting *SIR2*, *3 *or *4 *were limited to just the repressive chromatin features. The deletion of *SIR1 *produced no discernable differences to the chromatin structure upstream of the *URA3 *gene at XIL (the left panel in Figure 2B) in accord with the minor effect on the silencing levels seen in this strain (Figure 2A). The deletion of *SIR2*, *3 *or *4 *also produced a reduction in the minimal silencing seen at IIIR (Figure 2A). However, there were no detectable differences in the pattern of MNase-sensitive sites around or upstream of the *URA3 *promoter at IIIR in any of the three strains (the right panels in Figure 2B).

An analysis of the chromatin downstream of the *URA3 *gene in the *sir2, 3 or 4 *mutants revealed a prominent hypersensitive site at the 3' of the *URA3-GFP *construct (the asterisk in Additional File 3). This hypersensitive site may reflect a complete derepression of the reporter, since this site is also present at the 3' of the *URA3 *gene at its native locus [19]. The chromatin over core X was not altered at XIL or IIIR in any of the *sir *mutants (Additional File 3), in agreement with results obtained for telomere IIIL [21], which confirms that this structure does not reflect telomere silencing state.

### *yKU80 *is involved in the repressive chromatin structure at XIL

In addition to its roles in DNA repair and telomere maintenance, the yeast Ku heterodimer, composed of the yKu70 and yKu80 subunits, is involved in telomere position effect (TPE) [23-25]. In the absence of yKu, TPE is disrupted and less Sir3 and Sir4 are present in the subtelomeric regions [25-27], indicating that yKu is required in order to facilitate either the recruitment of the Sir complex or the assembly of a silencing competent structure. yKu is likely to play a direct role in TPE through an interaction with Sir4 [27].

**Figure 3:**
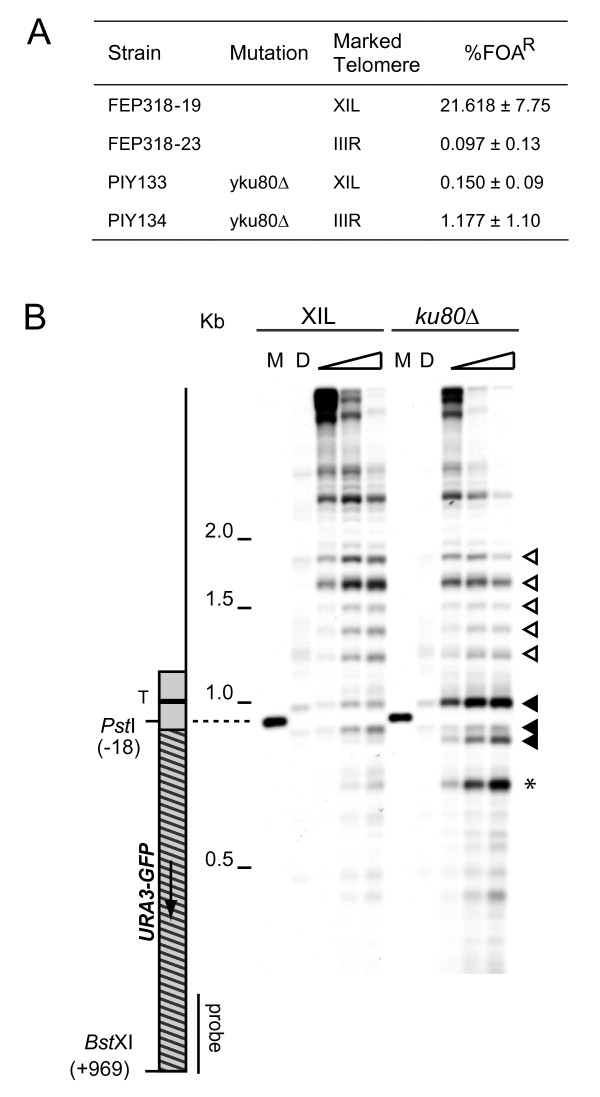
**Chromatin alterations towards the XIL centromere in a *yku80 *strain**. (A) Frequency of fluoroorotic acid (FOA) resistance in Δ*yku80::KanMX *strains containing the *URA3-yEGFP *marker adjacent to the core X element at the indicated telomere. The mean and standard deviation of FOA resistance is given for each strain. (B) The subtelomeric chromatin structure of the XIL telomere was analysed in the Δ*yku80::KanMX *strain, by MNase digestion and indirect end labelling, using a probe adjacent to the *BstX*I site within *yEGFP*. The marker (M) was generated by digestion of purified DNA with *BstX*I and *Pst*I. An MNase hypersensitive site within the reporter open reading frame is indicated by an asterisk, other labelling is as for Figure 1B.

Our previous work showed that the deletion of *yKU70 *causes a substantial reduction of silencing at both the truncated VIIL and the native XIL telomeres [17], and we have obtained similar results at XIL in a *yku80*Δ strain (Figure 3A). The chromatin structure toward the centromere is significantly altered at XIL in the *yku80*Δ strain (Figure 3B). The pattern of MNase sensitivity at the *URA3 *promoter resembles the open structure observed at IIIR (see Figure 1B) and the heterochromatic banding pattern, although intact, shows reduced band intensity (the white arrow heads in Figure 3B). There were also alterations to the chromatin over the *URA3 *open reading frame. Notably, a hypersensitive site was present in the 5' region of the gene that was absent from the control strain (asterisk in Figure 3B). This band is likely to reflect the unsilenced chromatin structure of this gene, as it was also present when the *URA3 *reporter was present at the native *URA3 *locus or the IIIR telomere (Figure 1B). No detectable differences were found in the chromatin structure over the core X element and towards the telomere at XIL or at the non-repressive end IIIR (results not shown). Thus, the deletion of *yKU80*, like *SIR2*, *3 *or *4*, appears to affect only the repressive chromatin features.

### Mutation of the ACS and Abf1 binding sites alters core X chromatin structure

**Figure 4:**
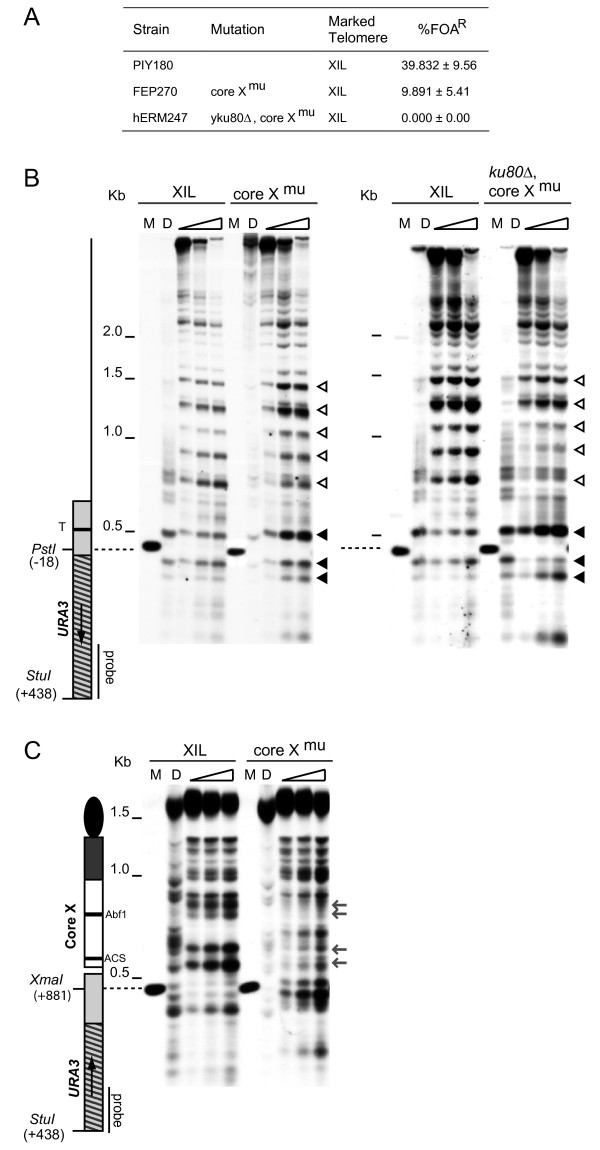
**Chromatin alterations at the XIL telomere in core X mutants**. (A) Frequency of fluoroorotic acid (FOA) resistance in strains containing the *URA3 *marker adjacent to the core X element at the XIL telomere. The core X^mut ^and core X^mut^, Δ*yku80::KanMX *strains contain mutations of the ARS consensus sequence (ACS) and Abf1 binding sites at the XIL core X element. The mean and standard deviation of FOA resistance is given for each strain. (B & C) Chromatin structure analysis of the XIL subtelomere in the core X^mut ^and core X^mut^, Δ*yku80::KanMX *strains was performed as described for Figure 1B and 1C. A schematic of the XIL telomere is shown, with black bars indicating the core X ACS and Abf1 binding sites. MNase hypersensitive sites that are altered in the core X^mut ^strain are indicated by grey arrows.

We have previously shown that mutation of either the ACS or the Abf1 binding site within a core X element causes moderate derepression of a *URA3 *reporter present at that end [17]. In order to determine whether or not these two binding sites also influence the surrounding chromatin structure, we constructed a strain that has the *URA3 *marker and both core X binding sites mutated at the XIL telomere. Disruption of both core X binding sites reduced repression at XIL by approximately fourfold (Figure 4A). The MNase digestion pattern toward the centromere shows a few significant changes in the core X mutant compared to the control strain (Figure 4B). The top and bottom promoter associated bands (the black arrow heads in Figure 4B) show an increased sensitivity to MNase, relative to the central band, indicating that the promoter was more accessible. However, the evenly spaced hypersensitive sites have a similar pattern in both the core X mutant and the control strain (the black arrow heads in Figure 4B).

More dramatic changes in the chromatin structure of the core X mutant were observed towards the telomere (Figure 4C). Four strong hypersensitive regions adjacent to the binding sites of ORC and Abf1 (the grey arrows in Figure 4C) were missing in the core X mutant and the remainder of the pattern over the core X element is similar to the deproteinized DNA pattern, indicating that the loss of the two binding sites results in the loss of the specialized chromatin structure over core X.

The deletion of *yKU80 *in a strain containing the mutated core X binding sites had a more pronounced effect on the chromatin structure at XIL than either of the single mutants (the right panel in Figure 4B). The top and bottom promoter proximal bands show increased cleavage, indicating that the promoter is in an open conformation (the black arrow heads in the right panel of Figure 4B), consistent with the loss of silencing in this strain (Figure 4A). The regularly spaced bands upstream of the promoter show a reduced MNase sensitivity and there is increased cleavage between the bands (the white arrow heads in the right panel of Figure 4B) similar to the pattern observed in the *SIR2*, *3 *or *4 *deletion strains (Figure 2B).

### Different histone modification requirements for silencing and nucleosome positioning at telomeres

Histone methylation, acetylation and the presence of the variant histone H2A.Z have all been proposed as defining euchromatic DNA or the boundary between euchromatin and heterochromatin [8,9,11,28]. The deletion of the proteins required to produce these modifications both perturbs telomere silencing and allows the Sir proteins to associate within euchromatin [9-11,28,29]. Since deletions of *SIR2*, *3 *or *4 *disrupted the heterochromatic features at the XIL telomere, it is possible that deletions of histone modifying proteins could indirectly affect the repressive chromatin by altering the distribution of the Sir proteins.

**Figure 5:**
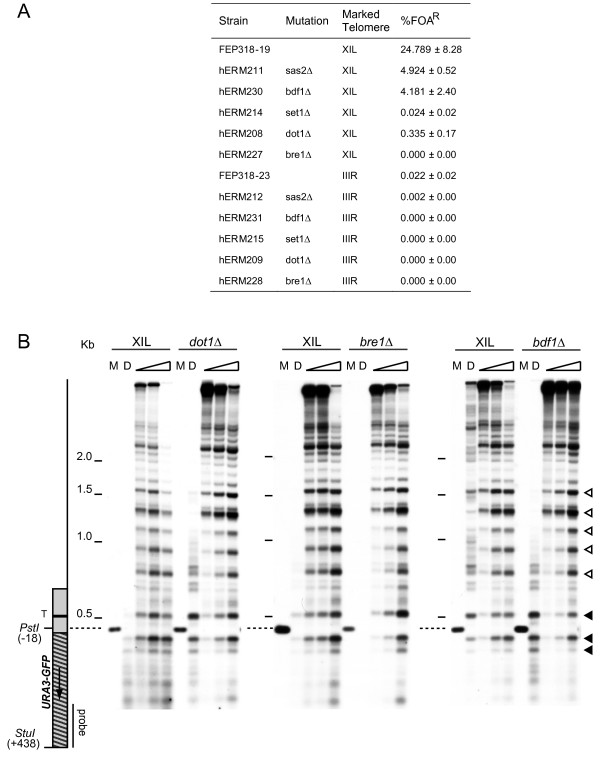
**Histone modifiers are required for silencing but not nucleosome positioning at telomeres**. (A) Frequency of fluoroorotic acid (FOA) resistance in isogenic Δ*bre1::KanMX*, Δ*dot1::KanMX*, Δ*set1::KanMX*, Δ*sas2::KanMX*, and Δ*bdf1::KanMX *strains containing the *URA3-yEGFP *marker adjacent to the core X element at the indicated telomere. The mean and standard deviation of FOA resistance is given for each strain. (B) The subtelomeric chromatin structure of the XIL telomere was analysed in the Δ*bre1::KanMX*, Δ*dot1::KanMX*, and Δ*bdf1::KanMX *strains, by MNase digestion and indirect end labelling, as for Figure 1B.

We constructed strains with gene deletions for five chromatin modification proteins; Bre1 an essential co-factor in the ubiquitination of lysine 123 on histone H2B [30], a modification required for the methylation of histone H3 lysine 4 by Set1 and lysine 79 by Dot1 [30-34]; Sas2 an acetyltransferase that targets lysine 16 on histone H4 [9]; and Bdf1 which is associated with the SWR1 complex that deposits H2A.Z [35-37]. Deletions of these genes are known to either reduce the expression of telomere proximal genes [9,11,28,38] or reduce silencing at truncated telomeres [30,31,33] and all five produced large reductions in silencing in our strains (Figure 5A). Chromatin structure analysis in these deletion strains revealed few alterations to the repressive features on the centromere-proximal side of core X at XIL (Figure 5B). The top promoter-associated band was slightly more sensitive to MNase in the *bre1*Δ and *dot1*Δ strains than in the control strain (the top black arrow head in Figure 5B), however the heterochromatic banding pattern remained intact. An analysis of the band intensity profiles by Kodak 1D scan software confirmed the absence of changes to the heterochromatic banding pattern at XIL (data not shown). An analysis of the chromatin structure over the core X and associated repeat elements at XIL showed no changes in the five deletion strains (Additional File 4). In addition, we did not observe any changes to the subtelomeric chromatin structure at the non-repressive telomere IIIR in any of the five deletion strains (data not shown). Therefore, despite the dramatic reductions to telomere silencing, the deletion of any of these five genes did not alter nucleosome positioning in the subtelomeres.

## Discussion

In order to understand how chromatin structure influences telomere silencing, we have analysed the chromatin structure of native ends that exhibit different silencing states. There were no differences detected between the repressive and non-repressive ends over core X towards the telomere. However, on the centromere proximal side of core X, we detected chromatin features that correlate with the TPE state and identify certain key factors that are necessary for repressive chromatin at telomeres.

Repressive ends exhibited a regular array of phased nucleosomes over the native subtelomeric sequence, similar to previously observed structures at other silenced regions such as *HML*, *HMR *[13,14] and the left end of chromosome III [21]. This phased nucleosome array is at a distance from the telomere, separated from it by the core X and X-associated repeat sequences found at the native ends. This chromatin structure is consistent with a fold-back model, previously proposed [17], in which the telomere physically interacts with the core X element, while the sequences in between loop-out and do not become involved in Sir-dependent silencing. The chromatin structure of the native repressive ends differs from that of truncated ends, which have a single continuous nucleosome array right up to the telomeric repeats embedded within a telosome [15]. In contrast to the repressed chromatin, each non-repressive end has a unique euchromatic structure over the region centromere proximal to core X.

We also detected chromatin differences among telomeres around the promoter region of the *URA3 *marker. At non-repressive and truncated telomeres the *URA3 *promoter region closely resembles its conformation when at its native location on chromosome V. Six nucleosomes are positioned across the *URA3 *gene at its native locus [19]. The first nucleosome is positioned immediately to the 3' of the *URA3 *TATA box, encompassing part of the promoter region and the first ~70 bp of the *URA3 *coding sequence [19]. At non-repressive telomeres we found a nucleosome similarly positioned adjacent to the 3' side of the *URA3 *TATA box. However, at telomeres where *URA3 *is repressed, the MNase sensitivity pattern indicates that the *URA3 *TATA box is less accessible and that the nucleosome positions have shifted, which we propose represents a closed promoter conformation.

The deletion of the Sir proteins 2, 3 or 4 had a large effect on the chromatin structure at the repressive end as expected. However, this was limited to the 'repressive features' and did not alter the chromatin structure of the core X element. The absence of the phased nucleosome array could be caused by a loss of nucleosomes or simply a loss of phasing. Either way the characteristic repressive chromatin pattern is abolished in the absence of Sir proteins. Consistent with the idea that non-repressive ends are euchromatic, there was no change in the chromatin at these ends in the absence of Sir proteins. This is also in agreement with the limited data available (due to lack of unique sequences) for Sir protein associations with specific subtelomeres [39]. The non-repressive telomere IIIR, which is unaffected by the deletion of *SIR2*, *3 *or *4*, has no detectable binding or association of these proteins adjacent to the telomere. However, Sir protein associations were detected at the silenced end XIL which exhibits the Sir-dependent chromatin structure [39].

The chromatin structure over a core X element and the X associated repeats has been described previously [21]. We have shown that the same structure is present over the core X and repeat elements at other ends irrespective of TPE state. This structure appears to be determined by the protein factors that bind to core X, because mutating the Abf1 and ACS binding sites within core X disrupts the structure.

Mutation of the Abf1 and ACS sites at core X also reduces TPE at particular ends [17] and we show here that these mutations alter the chromatin structure around the promoter of a *URA3 *marker adjacent to core X. The phased array of nucleosomes proximal to the promoter is still intact, though they are not quite as sharply demarcated. This demonstrates that the phased nucleosomes are not sufficient for silencing but are probably necessary for the silencing to occur. A similar change over the promoter chromatin is seen when yKu is deleted. In this mutant, TPE is abrogated and the phased nucleosomes again remain intact. It is possible that, in both cases, there is sufficient recruitment of Sir proteins to the region to produce the phased nucleosomes but there are either not enough Sir proteins to produce full repression or another factor is missing. Both yKu and core X could influence Sir protein recruitment to the region. The ORC protein when bound to the ACS site at core X could recruit the Sir complex via an interaction with Sir1, similar to its role at the *HM *silencers. yKu has been shown to associate with core X elements [40] and could recruit the Sir complex through a direct interaction with Sir4 [27,41]. Combining the y*ku80 *deletion with the core X mutations resulted in a loss of the phased nucleosomes, similar to that seen in the absence of Sir proteins. Recruitment of the Sir complex by yKu and by the factors binding to core X may, therefore, be independent of one another.

Histone methylation, acetylation, and H2A.Z incorporation have all been proposed to prevent the spread of the Sir proteins into euchromatin [8,9,11,28]. We deleted the genes for five different histone modifying proteins and, significantly, none of the deletions affected nucleosome positioning at the repressive telomere despite their causing a significant reduction in silencing at that end. Deletions of Sas2 and Dot1 reduce the concentration of Sir proteins in telomere proximal regions which may be responsible for the silencing defects of these strains [8]. Again, it is possible that a lower concentration of Sir proteins at the subtelomeres is sufficient to organize a phased nucleosome structure, but insufficient for full silencing.

**Figure 6:**
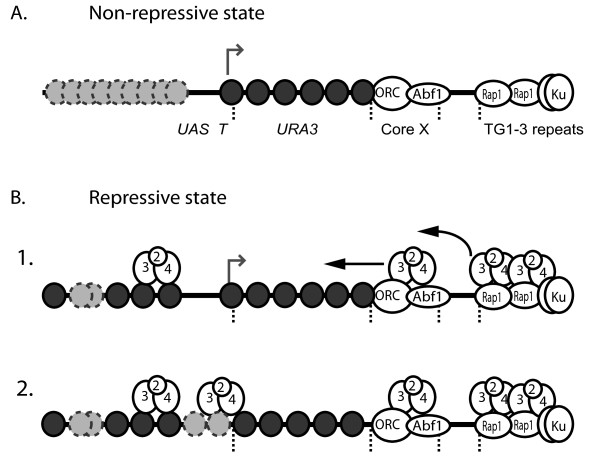
**Model of repressive and non-repressive chromatin at *Saccharomyces cerevisiae *telomeres**. Schematic shows a *URA3 *marked telomere with the positions of the *URA3 *coding sequence, upstream activating sequence (UAS), and TATA box (T) indicated. (A) The non-repressive state is characterized by an open promoter conformation and the presence of unpositioned nucleosomes (light grey circles) upstream of the *URA3 *promoter region. This structure permits transcription (grey arrow) of the *URA3 *gene. (B) The repressive state is formed in two steps: (1) Phased nucleosomes are positioned upstream of the *URA3 *gene by a mechanism dependent on the Sir proteins (2 - 4). Interaction of the Sir proteins with this upstream region could occur by looping or folding-back of the telomere (not depicted). (2) Full repression is established by an additional step, such as further Sir recruitment or histone modification, which results in the *URA3 *promoter assuming a closed chromatin structure.

Several previous studies have shown that, in particular conditions, Sir protein binding and spreading can occur without gene silencing [42-44]. We extend these results to show that Sir-dependent nucleosome positioning can occur without silencing. A similar result has also been obtained when a histone acetyltransferase was tethered within a region of silenced chromatin; in this case repression of a *URA3 *reporter gene was reduced without altering nucleosome positioning or removal of Sir proteins [45]. We suggest that the formation of telomeric heterochromatin occurs in several steps (Figure 6). In the first step Sir protein recruitment and spreading occurs, followed by Sir-dependent nucleosome positioning. Sir recruitment to the vicinity of core X is facilitated by both yKu and the core X element, possibly via a looping-back of the telomere. Full repression is achieved after a final step(s), which could involve further Sir protein recruitment, conformational changes to the Sir-chromatin structure, or histone modification.

## Methods

### Yeast strains and plasmids

All yeast strains were derived from the isogenic S288c strains FY833, 1679-13B, DS288c3-1a, and DS288C3-39c [46] or FYBL1-8B [47] and are listed in Additional File 5. Strains containing the core X mutations and the *URA3 *reporter construct adjacent to the core X of XIL were created as described previously [17]. Strains containing the *URA3-yEGFP *reporter construct adjacent to the core X of XIL and IIIR were obtained by transformation of FYBL1-8B [47] with the plasmid pFEP43 digested with *Sac*I. Strains containing the *URA3-yEGFP *reporter construct adjacent to a terminal truncation of VIIL were created by transformation of FYBL1-8B [47] with pFEP41 digested with *Eco*RI and *Sal*I. PIY125 was created by transforming DS288C3-39c with a PCR fragment containing the *URA3-yEGFP *cassette amplified from pFEP43 (using primers AAGAAACATGAAATTGCCCAG and AATTTGTGAGTTTAGTATACATGCATTTACTTATAATACAGTTTTTTATTTGTACAATTCATCCATAC). Gene deletions were created by replacement of coding sequences with the *kanMX4 *or *hphMX4 *cassettes [48,49].

Plasmids pFEP37 and pFEP40 were obtained by introducing an *Asc*I restriction site, by polymerase chain reaction mutagenesis, at the 3' end of the *URA3 *gene on plasmids pFEP24 [17] and pADH-UCAIV [20]. The *yEGFP3 *gene was amplified from pUG35 (provided by J Hegemann and U Güldener), using primers 5' GGCgcGCcCGTCGACCTCGACATGTCTA 3' and 5' ggcgcgccTTTGTACAATTCATCCATAC 3' and cloned into the *Asc*I site of pFEP37 and pFEP40 to create plasmids pFEP43 and pFEP41.

### Measurement of telomere silencing

Single colonies from strains marked with a subtelomeric *URA3 *gene were resuspended in water and serial dilutions spotted onto complete synthetic media and medium containing 5-FOA. The percentage of colonies resistant to 5-FOA after three days growth at 30°C was determined.

### Chromatin analysis using MNase

Chromatin analysis of yeast cells using micrococcal nuclease I was performed as previously described [50-52]. Spheroplasts were prepared from 1.2 × 10^9 ^yeast cells using zymolyase 100T and permeabilized with the detergent NP-40. Chromatin from 2.0 × 10^8 ^permeabilized cells was digested with 1, 2.5 or 5 units/ml of MNase at 37°C for 4 min. An equivalent amount of purified DNA was digested with 5 units/ml of MNase for 35 s at 37°C, to yield the deproteinized DNA digestion patterns. Marker DNA was obtained by digesting purified DNA from the same cells with an appropriate restriction enzyme. All samples were purified and analysed by indirect end-labelling [53] by digestion with either *Stu*I or *Bst*XI. Digested samples were separated by agarose gel electrophoresis and transferred to nylon membranes. The MNase digestion pattern towards either the centromere or telomere was visualized with an appropriate 200 bp probe adjacent to the end-label digestion site. Probes were generated by radio-labelling gel-purified polymerase chain reaction fragments amplified from yeast genomic DNA.

### Interpretation of MNase digests

As MNase preferentially cuts the linker DNA between nucleosomes the presence of a protected region of sites (that are cut in the deproteinized DNA control), flanked by two hypersensitive sites ~150 bp apart, is consistent with the placement of a translationally positioned nucleosome. Other non-histone DNA-binding proteins may also protect regions of DNA, of varying size, from MNase digestion. However, in this study we interpret regions of protection of ~150 bp to imply the presence of bound nucleosomes.

Relative band intensities of MNase cleavage products were also determined using KODAK 1D image analysis software (Kodak) in order to assist the interpretation of chromatin blots.

## Abbreviations

ACS: ARS consensus consequence; ORC: origin recognition complex; MAT: mating type; TPE: telomere position effect.

## Competing interests

The authors declare that they have no competing interests.

## Authors' contributions

ERL and PWI contributed equally to this work. ERL, PWI and SS performed the MNase digestion and end-labelling experiments. FEP, PWI and ERL were involved in the yeast strain creation. ERL, PWI, NAK, JM and EJL all participated in the overall study design and planning. EJL and SS wrote the manuscript. All authors read and approved the final manuscript.

## Supplementary Material

Additional file 1 **Repressive and non-repressive ends have distinct subtelomeric chromatin structures**. MNase digestion and indirect end labelling was used to analyse (A & C) the non-repressive telomeres IVL and XVR and (B) the repressive telomere IIR, as described in Figure 1. The chromatin structure is shown (A & B) upstream of the *URA3 *marker or (C) downstream of the *URA3 *marker (towards the telomere). (A & B) Arrows adjacent to the blots indicate the most telomere-proximal open reading frames: YOR394W (e), YDL248W (f) and YBR302C (g). (C) A schematic of the XVR telomere is shown, with the core X ACS and Abf1p binding sites indicated by black bars. Telomere IVL is identical except that it lacks a Y' element. Click here for file

Additional file 2 **Integration of a *URA3 *marker does not alter the chromatin structure of telomere XIL**. (A) Schematic of telomere XIL showing positions of *URA3 *marker insertion (1 - 5). Position 1, adjacent to the core X ACS site, is the site used in the majority of the strains described in this paper. (B&C) The subtelomeric chromatin structure of XIL was analyzed by MNase digestion and indirect end labelling, as described in Figure 1, in strains that have a *URA3 *marker inserted into one of the locations shown in (A). Chromatin structure was analysed (B) upstream of the *URA3 *marker (towards the centromere) and (C) downstream of the *URA3 *marker (towards the telomere). Three hypersensitive sites associated with the *URA3 *promoter are indicated by black arrow heads and an array of evenly spaced hypersensitive sites by white arrow heads. MNase hypersensitive sites adjacent to the core X ACS and Abf1p binding sites are indicated by grey arrows. Click here for file

Additional file 3 **Deletion of Sir proteins does not alter the chromatin structure of core X**. Chromatin structures of the XIL and IIIR telomeres were analysed in Δ*sir1::KanMX*, Δ*sir2::KanM*, Δ*sir3::KanM *and Δ*sir4::KanM *strains, by MNase digestion and indirect end labelling, as described for Figure 1C. An MNase hypersensitive site at the terminus of the reporter gene is indicated by an asterisk. Click here for file

Additional file 4 **Histone modifiers are not required for nucleosome positioning at telomeres**. The subtelomeric chromatin structure of the XIL telomere was analysed in the Δ*bre1::KanMX*, Δ*dot1::KanMX *and Δ*bdf1::KanMX *strains, by MNase digestion and indirect end labelling, as for Figure 1C. Click here for file

Additional file 5 Yeast strains used in this study. Click here for file
