# Supplementary figures and images for: Repressive and non-repressive chromatin at native telomeres in Saccharomyces cerevisiae
Source: Epigenetics Chromatin. 2009 Dec 2;2:18. doi: 10.1186/1756-8935-2-18 (PMC3225887; doi:10.1186/1756-8935-2-18)

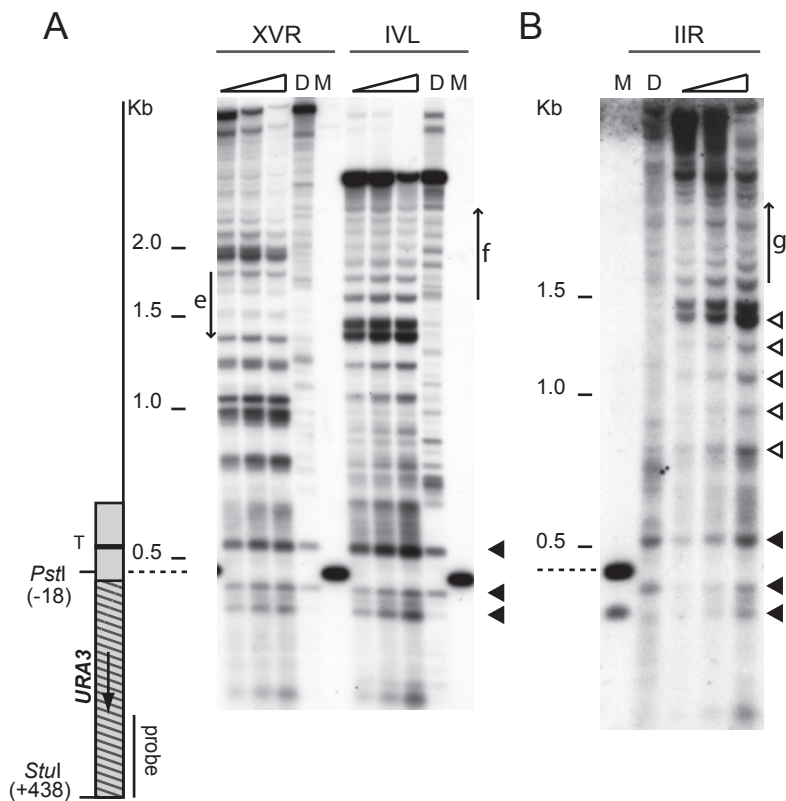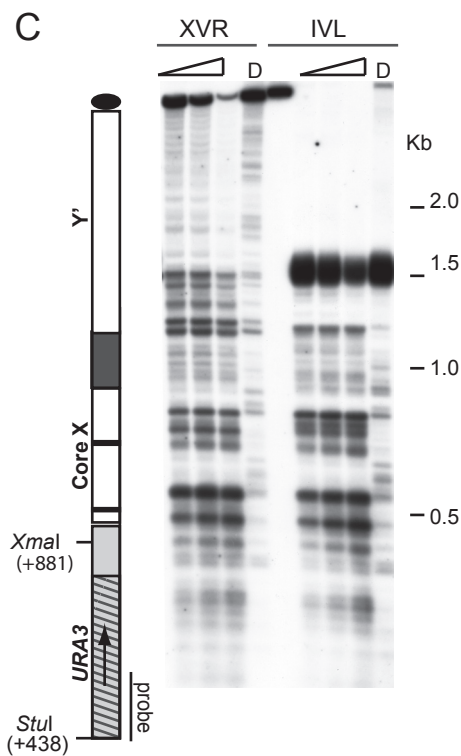

Supplement: Additional file 1 — Repressive and non-repressive ends have distinct subtelomeric chromatin structures. MNase digestion and indirect end labelling was used to analyse (A & C) the non-repressive telomeres IVL and XVR and (B) the repressive telomere IIR, as described in Figure 1. The chromatin structure is shown (A & B) upstream of the URA3 marker or (C) downstream of the URA3 marker (towards the telomere). (A & B) Arrows adjacent to the blots indicate the most telomere-proximal open reading frames: YOR394W (e), YDL248W (f) and YBR302C (g). (C) A schematic of the XVR telomere is shown, with the core X ACS and Abf1p binding sites indicated by black bars. Telomere IVL is identical except that it lacks a Y' element. [file 1756-8935-2-18-S1.PDF]

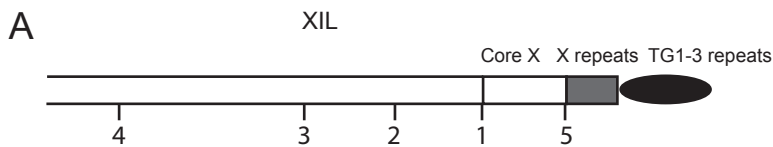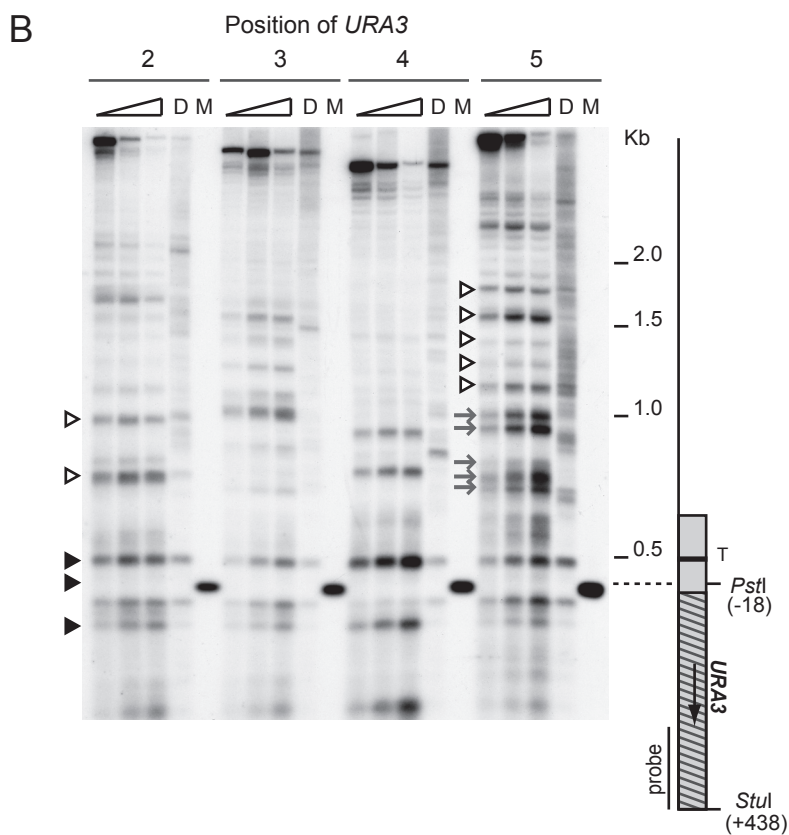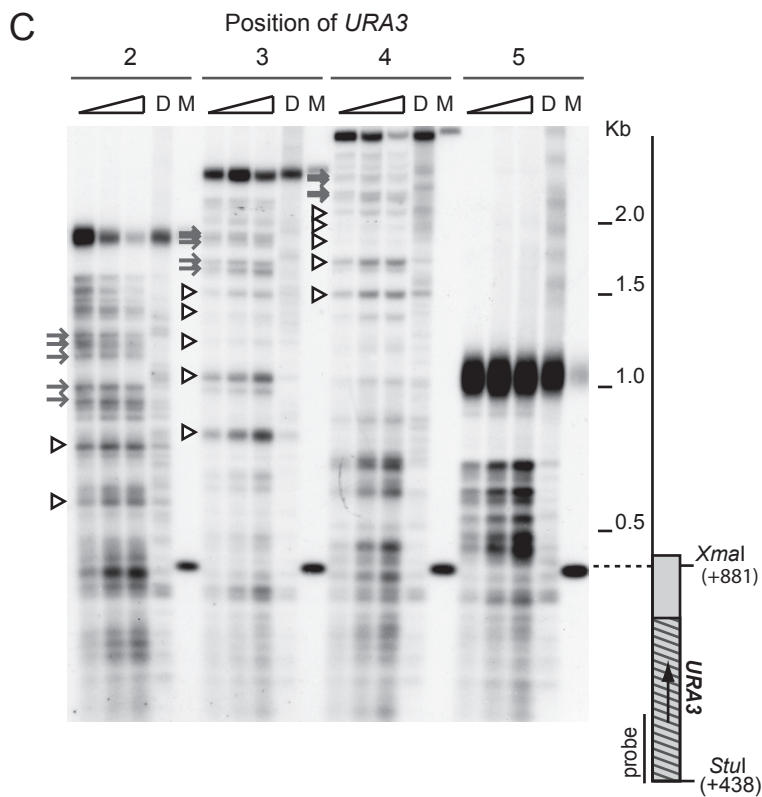

Supplement: Additional file 2 — Integration of a URA3 marker does not alter the chromatin structure of telomere XIL. (A) Schematic of telomere XIL showing positions of URA3 marker insertion (1 - 5). Position 1, adjacent to the core X ACS site, is the site used in the majority of the strains described in this paper. (B&C) The subtelomeric chromatin structure of XIL was analyzed by MNase digestion and indirect end labelling, as described in Figure 1, in strains that have a URA3 marker inserted into one of the locations shown in (A). Chromatin structure was analysed (B) upstream of the URA3 marker (towards the centromere) and (C) downstream of the URA3 marker (towards the telomere). Three hypersensitive sites associated with the URA3 promoter are indicated by black arrow heads and an array of evenly spaced hypersensitive sites by white arrow heads. MNase hypersensitive sites adjacent to the core X ACS and Abf1p binding sites are indicated by grey arrows. [file 1756-8935-2-18-S2.PDF]

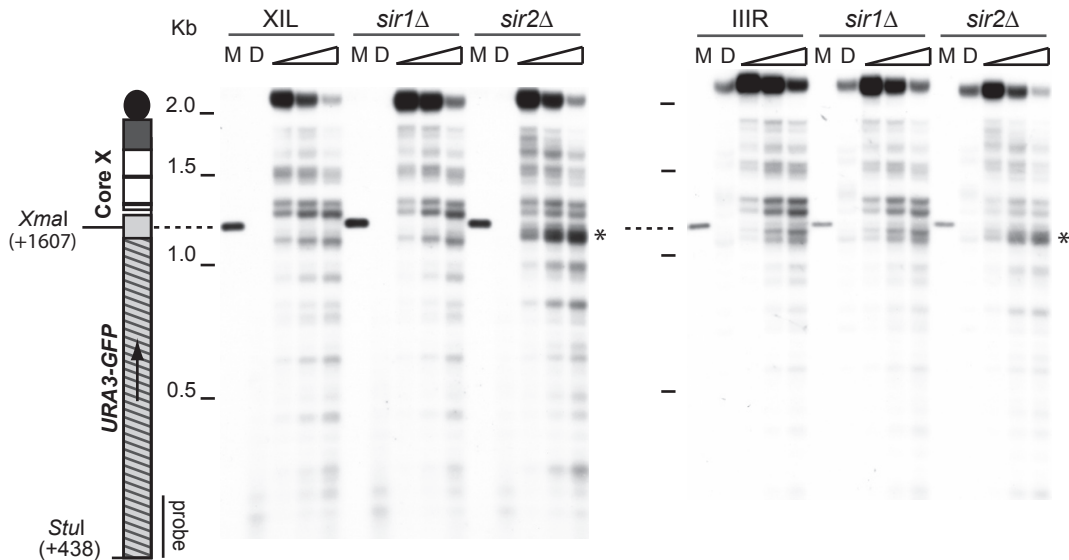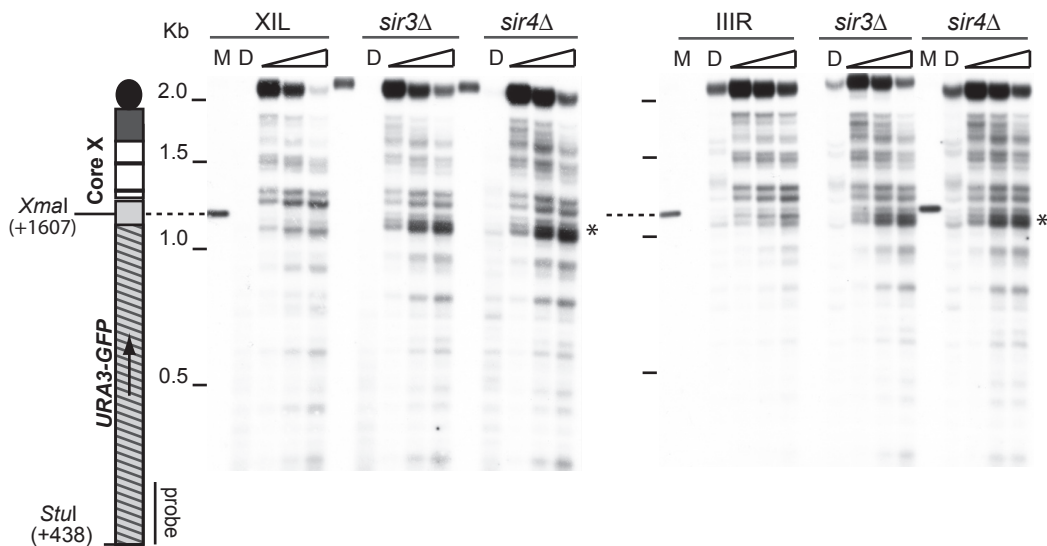

Supplement: Additional file 3 — Deletion of Sir proteins does not alter the chromatin structure of core X. Chromatin structures of the XIL and IIIR telomeres were analysed in Δsir1::KanMX, Δsir2::KanM, Δsir3::KanM and Δsir4::KanM strains, by MNase digestion and indirect end labelling, as described for Figure 1C. An MNase hypersensitive site at the terminus of the reporter gene is indicated by an asterisk. [file 1756-8935-2-18-S3.PDF]

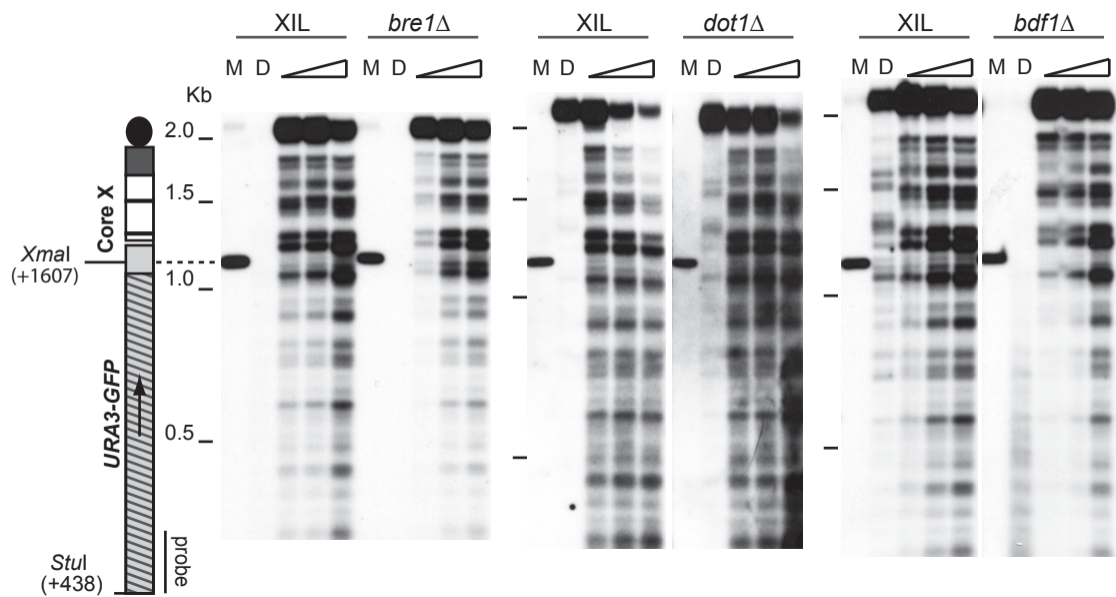

Supplement: Additional file 4 — Histone modifiers are not required for nucleosome positioning at telomeres. The subtelomeric chromatin structure of the XIL telomere was analysed in the Δbre1::KanMX, Δdot1::KanMX and Δbdf1::KanMX strains, by MNase digestion and indirect end labelling, as for Figure 1C. [file 1756-8935-2-18-S4.PDF]
