# Supplementary material for: Repressive and non-repressive chromatin at native telomeres in Saccharomyces cerevisiae
Source: Epigenetics Chromatin. 2009 Dec 2;2:18. doi: 10.1186/1756-8935-2-18 (PMC3225887; doi:10.1186/1756-8935-2-18)
Supplement: Additional file 5 — Yeast strains used in this study. [file 1756-8935-2-18-S5.DOC]

| Strain | Genotype | Reference |
| --- | --- | --- |
| FYBL1-8B | *MAT****a*** *lys2∆202 leu2∆1 his3∆200 ura3∆851* | Fairhead et al., 1996 |
| FEP100-10 | *MAT****a*** *leu2∆1 ura3-52 can1-1 ade2∆ URA3-*TELXIL | Pryde & Louis 1999 |
| FEP175 | FYBL1-8B *URA3-*TELXVR | Pryde & Louis 1999 |
| FEP180 | FYBL1-8B *URA3-*TELXIL position 2 | Pryde & Louis 1999 |
| FEP184 | FYBL1-8B *URA3-*TELXIL position 3 | Pryde & Louis 1999 |
| FEP193 | FYBL1-8B *URA3-*TELXIL position 4 | Pryde & Louis 1999 |
| FEP210 | FYBL1-8B *URA3-*TELXIL position 5 | Pryde & Louis 1999 |
| FEP229-4 | FYBL1-8B *URA3-*TELIVL | Pryde & Louis 1999 |
| FEP229-13 | FYBL1-8B *URA3-*TELIIR | Pryde & Louis 1999 |
| FEP270-1 | FYBL1-8B *URA3-*TELXIL ACS-*NdeI*-TELXIL Abf1-*SphI-*TELXIL | This study |
| FEP318-19 | FYBL1-8B *URA3-yEGFP-*TELXIL | This study |
| FEP318-23 | FYBL1-8B *URA3-yEGFP-*TELIIIR | This study |
| hERL3 | *MAT****a*** *lys2∆202 leu2∆1 ura3-52::hphMX4 adh4*::*URA3-yEGFP-*TELVIIL | This study |
| hERL5 | FYBL1-8B *URA3-yEGFP-*TELXIL *sir1::kanMX* | This study |
| hERL6 | FYBL1-8B *URA3-yEGFP-*TELIIIR *sir1::kanMX* | This study |
| hERL7 | FYBL1-8B *URA3-yEGFP-*TELXIL *sir2::kanMX* | This study |
| hERL8 | FYBL1-8B *URA3-yEGFP-*TELIIIR *sir2::kanMX* | This study |
| hERL9 | FYBL1-8B *URA3-yEGFP-*TELXIL *sir3::kanMX* | This study |
| hERL10 | FYBL1-8B *URA3-yEGFP-*TELIIIR *sir3::kanMX* | This study |
| hERL11 | FYBL1-8B *URA3-yEGFP-*TELXIL *sir4::kanMX* | This study |
| hERL12 | FYBL1-8B *URA3-yEGFP-*TELIIIR *sir4::kanMX* | This study |
| hERM208 | FYBL1-8B *URA3-yEGFP-*TELXIL *dot1::kanMX* | This study |
| hERM209 | FYBL1-8B *URA3-yEGFP-*TELIIIR *dot1::kanMX* | This study |
| hERM211 | FYBL1-8B *URA3-yEGFP-*TELXIL *sas2::kanMX* | This study |
| hERM212 | FYBL1-8B *URA3-yEGFP-*TELIIIR *sas2::kanMX* | This study |
| hERM214 | FYBL1-8B *URA3-yEGFP-*TELXIL *set1::kanMX* | This study |
| hERM215 | FYBL1-8B *URA3-yEGFP-*TELIIIR *set1::kanMX* | This study |
| hERM227 | FYBL1-8B *URA3-yEGFP-*TELXIL *bre1::kanMX* | This study |
| hERM228 | FYBL1-8B *URA3-yEGFP-*TELIIIR *bre1::kanMX* | This study |
| hERM230 | FYBL1-8B *URA3-yEGFP-*TELXIL *bfd1::kanMX* | This study |
| hERM231 | FYBL1-8B *URA3-yEGFP-*TELIIIR *bdf1::kanMX* | This study |
| hERM238 | FYBL1-8B *URA3-yEGFP-*TELXIL *yku70::kanMX* | This study |
| hERM239 | FYBL1-8B *URA3-yEGFP-*TELIIIR *yku70::kanMX* | This study |
| hERM247 | FYBL1-8B *URA3-*TELXIL ACS-*NdeI-*TELXIL Abf-*SphI-*TELXIL *yku80::kanMX* | This study |
| PIY125 | *MATα* *lys2∆202 leu2∆1 his3∆200 ura3-52::URA3-yEGFP* | This study |
| PIY133 | *MAT****a*** *lys2∆202 leu2∆1 his3∆200 ura3-52 URA3-yEGFP-*TELXIL *yku80::kanMX* | This study |
| PIY134 | *MATα* *lys2∆202 leu2∆1 his3∆200 ura3∆851 URA3-yEGFP-*TELIIIR *yku80::kanMX* | This study |
| PIY180 | FEP100-10 *ura3-52::kanMX* | This study |
